# Fetal and Postnatal Nicotine Exposure Modifies Maturation of Gonocytes to Spermatogonia in Mice

**DOI:** 10.1155/2020/8892217

**Published:** 2020-12-15

**Authors:** Rosa María Vigueras-Villaseñor, Martín Alejandro Fuentes-Cano, Margarita Chávez Saldaña, Liliana Rivera Espinosa, Rafael Reynoso-Robles, Patricia Rojas, Pilar Durán, Julio César Rojas-Castañeda

**Affiliations:** ^1^Laboratorio de Biología de la Reproducción, Instituto Nacional de Pediatría, SS, Mexico City 04530, Mexico; ^2^Laboratorio de Biología Animal Experimental, Facultad de Ciencias, UNAM, Mexico City 04510, Mexico; ^3^Laboratorio de Farmacología, Instituto Nacional de Pediatría, SS, Mexico City 04530, Mexico; ^4^Laboratorio de Morfología Celular y Tisular, Instituto Nacional de Pediatría, SS, Mexico City 04530, Mexico; ^5^Laboratorio de Neurotoxicología, Instituto Nacional de Neurología y Neurocirugía, “Manuel Velasco Suárez”, SS, Mexico City 14269, Mexico

## Abstract

Studies in laboratory animals have shown that male offspring from dams, exposed to nicotine during pregnancy and postnatal periods, show alterations in fertility, although the origin of this is still uncertain. In this study, we examined in a mouse model if the process of gonocyte maturation to spermatogonia was affected in male offspring from dams with nicotine administration during pregnancy and postnatal periods. BALB/C mice, with and without nicotine administrations in pregnancy and postnatal periods, were studied. The animals were euthanized at 3, 7, 10, 16, and 35 days postpartum (dpp). Testicular tissue samples were processed for histological, ultrastructural, and immunohistochemical studies; and testicular lipoperoxidation was determined. It was observed that in the nicotine-exposed animals, there was increased apoptosis and a reduction in the number of gonocytes that matured to spermatogonia. This gonocyte-spermatogonia maturation reduction was associated with a greater immunoreactivity to nicotinic acetylcholine receptors in the germ cells. Lipoperoxidation was similar in both groups until 16 dpp, with significant reduction at 35 dpp. Our findings suggest that nicotine intake during pregnancy and postnatal periods can affect the process of maturation of gonocytes to spermatogonia and the pool of available spermatogonia for spermatogenesis.

## 1. Introduction

The World Health Organization report on smoking revealed that one-third of the world population are smokers. Cigarette has a variety of components including nicotine. Nicotine is a toxic alkaloid with a high addictive potential. It has the ability to cross through the placental barrier and to permeate into maternal milk of smoking mothers [1, 2]. In addition, nicotine is associated with child mortality as well as premature births, abortion risk, and low birth weight that are associated with increased morbidity [3].

There are several studies showing the effect of nicotine exposure on male adult reproductive ability [4]. Also, the fertile capacity of sons from smoking mothers who indulge in smoking during pregnancy and lactation is affected [5–11].

The number of experimental studies using animal models and showing fertility alterations in offspring exposed to nicotine during pregnancy and lactation is scarce [12–16]. These studies were centered on offspring in adult stage. Their results depicted different alterations, such as low sperm count and decreased spermatogenesis and fecundability as well as reduced number of morphologically normal sperm cells [14]. However, the possible mechanisms of nicotine action on the maturation process of gonocytes to spermatogonia in the neonatal period, which is crucial for a successful fertility, are not known. The gonocytes arise during the embryonic development, a time when the primordial germ cells migrate to the gonadal crest. Gonocytes are located in the center of the seminiferous cords. In humans, their maturation to spermatogonia, in this place, begins at the tail end of the third period of gestation and continues until the first six months of life. In mice, this occurs in the first six days postpartum (dpp) [17, 18]. During this process, the gonocytes usually migrate to the basement membrane and may or may not proliferate. This migration to the basement membrane is important for the survival of the gonocytes, since there is apoptotic death to those that remain in the center of the cord [19]. On the other hand, the number of spermatogonia is decisive for adequate sperm concentration. Nonetheless, the action of nicotine could be direct, because testicular tissue possesses nicotinic acetylcholine receptors (nAChR), and early germ cells of Sertoli and Leydig cells possess an *α*7 subunit of these receptors. It was suggested that nAChR might mediate cell divisions, metabolism, and motility in testicular tissue [20–22]. Furthermore, nicotine can have an indirect action, such as the generation of oxidative stress through either an increase in the concentrations of reactive species, deficiency of endogenous antioxidant system, [10, 23, 24] or altering the hormone concentrations [13, 16].

Therefore, the aim of this study was to determine, in a model of mice, whether the offspring from dams with exposition to nicotine during pregnancy and postnatal periods showed affectation in gonocyte survival, its proliferation, and maturation to spermatogonia, as well as to determine the participation of nAChR and lipoperoxidation in such process.

The results obtained indicated that nicotine intake during pregnancy and postnatal periods can affect the process of maturation of gonocytes to spermatogonia and the pool of available spermatogonia for spermatogenesis.

## 2. Materials and Methods

### 2.1. Animals

Albino mice BALB/c bred and housed at the vivarium of Facultad de Ciencias, UNAM, were maintained under standard colony conditions. Administration of food (Rodent Lab Chow 5001, Purina Inc.) and water was *ad libitum*. The animals were kept in a temperature- and light-controlled room (12 : 12 h light-dark cycle, temperature between 22 and 24°C, and humidity 40-50%). In order to obtain the experimental offspring, males of 25 g and females of 20 g were kept together overnight to mate, and the following morning, the females were separated and checked by inserting a vaginal tampon; this day was recorded as day 0 of gestation. Later, the animals were divided into two groups: control and nicotine exposed.

At birth, litters were standardized to 8 pups (6 males/2 females). We used only the male offspring, and these were maintained at the vivarium until sacrifice, with weaning at 21 dpp. Thirty male pups from each group (control and nicotine exposed) were assigned to five age subgroups (*n* = 6 each) as follows: (1) 3 dpp (the age when clear maturation of gonocytes to spermatogonia is observed); (2) 7 dpp (the age when the maturation of gonocytes to spermatogonia culminates with few or no gonocytes present); (3) 10 dpp (the age that later stages of spermatocytes appear beyond leptotene); (4) 16 dpp (the age when pachytene spermatocytes are detected), and (5) 35 dpp (the age of completion of spermatogenesis) [18].

All animals were treated in line with the ethical principles and regulations approved by the Animal Care and Use Committee (INV/B/RGC/107/18; Instituto Nacional de Pediatría, SS) and in accordance with Mexican NOM 062-200-1999, technical specifications for the reproduction, care, and use of laboratory animals (D.F. 22-VIII-01).

### 2.2. Nicotine Administration

Nicotine (Nic-Select® commercial trade used for electronic cigarette) was administered at 6 mg/kg/day to dams in drinking water *ad libitum* [25, 26] for 10 days before mating. The dose was maintained during mating, pregnancy, and lactation until weaning (21 dpp). Later, it was administered to pups in drinking water until 35 dpp. This method of administration was chosen to reduce manipulation stress [27]. The dose administered to the pups is equivalent to a heavy cigarette smoker who consumes from 1.5 to 3 packs/day [25, 26]. Water intake and body weight were recorded every 3 days throughout the exposure period. In addition, these data were used to adjust the nicotine quantity throughout the treatment.

All the mice were euthanized using an overdose of sodium pentobarbital (100 mg/kg, IP; Pfizer, Toluca, Estado de Mexico, Mexico) between 12:00 and 13:00 h with the objective of preventing circadian fluctuations. The testes were extracted, weighed, and washed in saline solution (0.9%). Each testis was half-sliced and placed on dry ice, immediately stored at -70°C, and used to determine lipoperoxidation. The other half was divided in parts—one for embedding in Epon 812 (Ted Pella, Inc., Redding, CA, USA) and the other for inclusion in paraffin.

### 2.3. Cotinine Concentration Determination

Cotinine is the major metabolite of nicotine with a longer half-life (approximately 20 h) than nicotine (20-60 min) [28]. A drop of peripheral blood from each animal was collected on Whatman 903® filter paper cards (GE Healthcare Bio-Sciences Corp; Piscataway, NJ, USA). Each card was horizontally dried for 6 h at room temperature (25 ± 1°C). Once dried, the cards were properly labelled and stored in a drying material packed in plastic bags with low gas permeability (-80°C) until analyzed. The entire circle was used for the quantification of cotinine. Ethyl acetate (Merck, Darmstadt, Germany) (1 mL), 10 *μ*L of ascorbic acid (Merck) (1%), and 10 *μ*L of 1% ammonium hydroxide (Merck) were used for extraction. Chromatographic separation was carried out using Acquity UPLC equipment with an XSelect HSS Cyano, 2.1 × 150 mm, 5 *μ*m (Waters®) adjusted to 40°C. The mobile phase consisted of 0.1% formic acid (Merck) in 5 mM ammonium formate (Sigma-Aldrich, St. Louis, MO, USA): acetonitrile (EMD Millipore Co®, Mexico) (50 : 50 *v*/*v*) at 0.3 mL/min. LC-MS/MS (Quattro Micro®; Waters Co.®, Milford MA, USA) was used for analysis. Detection was done by ESI^+^. Cotinine was measured in SRM mode, and ion transition was 177.26 > 80.14. Data were processed with MassLynx® 4.1 software. With the conditions described, the test was linear over the concentration range of cotinine 0.5 to 10 ng/mL.

### 2.4. Morphological Evaluation of the Gonocytes

Testicular tissue samples were fixed in modified Karnovsky solution without Ca^2+^ for 2 h. Later, they were postfixed in 1% OsO_4_ (Merck), dehydrated, and processed for embedding in Epon 812 (Ted Pella, Inc., Redding, CA, USA). Subsequently, the materials were sectioned at 1 *μ*m thick using an Ultracut UCT microtome (Leica, Vienna, Austria) and stained using 0.5% toluidine blue. The histological analysis of the seminiferous cords or tubules was performed using a BX 51 Olympus light microscope (Olympus Corp., Tokyo, Japan). Twenty to thirty transversal sections of the seminiferous cords or tubules per animal were evaluated. The area of seminiferous epithelium was determined by subtracting the internal area (tubular lumen) from the external area, using an image analyzing system (Image-Pro Plus 7.0, Media Cybernetics, Inc., MD, USA). The number of gonocytes (in contact or not) with the basement membrane and the number of spermatogonia were determined per seminiferous cord/tubule. The results were expressed per 1000 *μ*m^2^. Independent of the level of maturity of the gonocytes, we counted all those with evident nuclei in the section plane and classified them into types I, II, and III, as reported by Drumond et al. [18]. All histological examinations were performed by a single observer.

To confirm the presence of different types of gonocytes and their degeneration, the testicular tissues were sectioned at 60-70 nm thickness. Sections were stained with uranyl acetate and lead citrate and examined with a JEM-1011 (JEOL, Osaka, Japan) microscope.

### 2.5. Determination of Cell Proliferation and *α*7-nAChR

The testicular samples were fixed in 4% paraformaldehyde for 2 h, dehydrated, clarified, and embedded in paraffin. Sections of 4 *μ*m thickness were cut and mounted on slides with poly-l-lysine (Sigma-Aldrich, St. Louis, MO, USA). The tissue sections were deparaffinized with xylene and hydrated through a graded ethanol series. Later, the sections were exposed to citrate buffer (pH 7.6) for 5 min in a microwave oven set at 800 W. Then, the sections were delineated by a Dako pen (Dako, Carpinteria, CA, USA). The tissue sections were treated with 3% hydrogen peroxide for 10 min. Subsequently, they were placed in 0.1% Tween 20 (Sigma-Aldrich) phosphate-buffered saline (PBS, pH 7.4) solution for 10 min, blocked with 1% bovine serum albumin in PBS for 2 h, and incubated overnight with primary antibody. To determine cell proliferation and nicotine receptors, the sections were incubated at room temperature with rabbit-polyclonal antibodies against phospho-histone H3, (Millipore Upstate, MA, USA) and *α*7-nAChR (ABCAM, Cambridge, MA), at a dilution of 1 : 500 and 1 : 150, respectively. Sections were then incubated with biotinylated anti-rabbit IgG (Santa Cruz Biotechnology, CA USA) at a 1 : 100 dilution for 1 h and then with streptavidin-peroxidase conjugate (Rabbit ImmunoCruz staining system, Santa Cruz Biotechnology) for 30 min in accordance with the manufacturer's instructions. Tissue sections were incubated in a peroxidase substrate solution containing 1.6 mL of distilled H_2_O, 20 *μ*L 10x substrate buffer, and 40 *μ*L 50x diaminobenzidine chromogen (kit from Santa Cruz Biotechnology, CA, USA) and 1% H_2_O_2_ (Merck) in methanol for 30 min. Afterwards, they were counterstained with hematoxylin, dehydrated, and cleared with xylene. All dilutions and thorough washes between steps were performed using PBS unless otherwise specified. Negative control sections were processed in an identical manner but the primary antibodies were omitted. Tissue sections were mounted with Entellan mounting medium (Merck). The histological analysis of the seminiferous cords or tubules was performed by an observer with the help of a BX 51 Olympus light microscope. The number of proliferating germ cells was determined in twenty to thirty transversal sections of the seminiferous cords or tubules per animal and expressed per 1000 *μ*m^2^ tissue. All histological examinations were performed by a single observer.

### 2.6. Immunoreactivity (Optical Density) for *α*7-nAChR in Germ Cells

To determine variations in the protein expression at cellular level in the histological sections, we used optical density (OD). This is because OD has been used as a tool for indirect determination of the quantity of proteins in the histological sections. In addition, the results obtained with OD is similar to the results with biochemical techniques [29].

For OD analysis, digital images of tissue sections stained for *α*7-nAChR were captured at a magnification of ×100. Thirty well-delimited cellular bodies with cellular nucleus evident were randomly chosen and outlined manually for each animal to measure OD.

The OD measurements (expressed as arbitrary OD units in 10 *μ*m^2^) were automatically determined using an image system (Image-Pro Plus 7.0, Media Cybernetics, Inc., MD, USA). For each of the cells, two background OD measurements were determined in nearby regions without immunoreactive profiles. The mean background OD value calculated was subtracted from the cellular OD value measured to obtain the final OD value [30].

### 2.7. Apoptotic Cell Determination

To determine apoptosis, terminal deoxynucleotidyl transferase dUTP nick end labeling (TUNEL) technique was used (*in situ* Cell Death Detection Kit, Roche Diagnostic Corporation, Indianapolis, IN, USA). The testicular portions were fixed in 4% paraformaldehyde for 18 h. These tissue samples were dehydrated, clarified, and embedded in paraffin. Subsequently, a 4 *μ*m thickness was cut from each tissue sample and mounted on a slide covered with poly-l-lysine (Sigma-Aldrich). Next, they were deparaffinized and hydrated in a graded ethanol series. Sections were delineated with a Dako pen (Dako), treated with 0.1% Triton X-100 solution (Sigma-Aldrich) for 2 min, and then incubated in TUNEL solution (50 *μ*L terminal deoxynucleotidyl transferase and 450 *μ*L nucleotide mixture) for 1 h at 37°C. For staining specificity, we processed some sections through all the incubation steps and treated them with DNase (Stratagene) at a concentration of 1 *μ*g/1 mL for 10 min at 37°C, before incubation in the TUNEL solution, to induce DNA strand breaks. These tissue sections were mounted with Entellan (Merck) for observation using an Olympus fluorescence microscope (Olympus BX51). The number of apoptotic cells per seminiferous cord/tubule was calculated, and the results were expressed as the number of apoptotic cells per 1000 *μ*m^2^. Twenty to thirty transverse sections of the seminiferous tubules per animal were evaluated. All dilutions and washes between steps were performed using PBS (0.1 M) unless otherwise specified. Negative control sections were processed in an identical manner but the enzyme solution (terminal deoxynucleotidyl transferase) was omitted. Slides from different ages were randomized and coded in such a way that all subsequent analyses were conducted in a blinded manner. All histological examinations were performed by a single observer.

### 2.8. Lipoperoxidation through Thiobarbituric Acid-Reactive Substances

Production of thiobarbituric acid-reactive substances (TBARS) was calculated according to the modified technique described for *in vitro* studies [31]. Due to the reduced size of the testes of 3, 7, 10, and 16 dpp, we homogenized them in groups of nicotine and control per age. The testes of 35 dpp were homogenized for each animal. For each homogenized tissue, 1 mL was taken and added to 2 mL of the thiobarbituric acid (TBA) reagent (0.375 g of TBA, 15 g of trichloroacetic acid, and 2.5 mL of concentrated HCl in 100 mL of distilled water), and the final solution (3 mL total volume) was heated in a hot bath for 30 min. Samples were cooled and centrifuged at 3000 × g for 15 min. The absorbance was measured in supernatants by spectrophotometry at 532 nm. TBARS concentrations were calculated by the interpolation of a periodic oxidation of a malondialdehyde standard curve. The final result was expressed as nanomoles of TBARS per milligram of protein. Protein content in the testicular tissue samples was measured using the method of Lowry et al. [32]. The results of lipoperoxidation were normalized to the protein content in each sample.

### 2.9. Statistical Analysis

Data were expressed as median and interquartile ranges. Results were analyzed using Mann*-*Whitney *U* test, comparing the experimental group versus the control group per age group. Values of *p* < 0.05 were considered significant.

## 3. Results

### 3.1. Cotinine Concentration, Anatomy, Histology, and Lipoperoxidation

The presence of cotinine in blood was in all ages of the nicotine group, in contrast to what was observed in the control group (*p* < 0.05; Table 1).

At 3 and 16 dpp, the nicotine-exposed group presented a significantly reduced body weight, compared with the control group (*p* < 0.05). However, at 7, 10, and 35 dpp, no significant differences in body weight were observed in animals of these ages when compared with the control group (*p* > 0.05, Table 1). When testicular weight was analyzed in relation with body weight, there was no statistically significant difference in any of the age groups when compared with the controls (*p* > 0.05, Table 1).

Of the six animals with nicotine administration studied of 35 dpp, only one developed bilateral inguinal cryptorchidism. This animal was eliminated from histological and immunohistochemical studies, since cryptorchidism generates testicular histological alterations.

#### 3.1.1. Animals of 3 dpp

**Figure 1:**
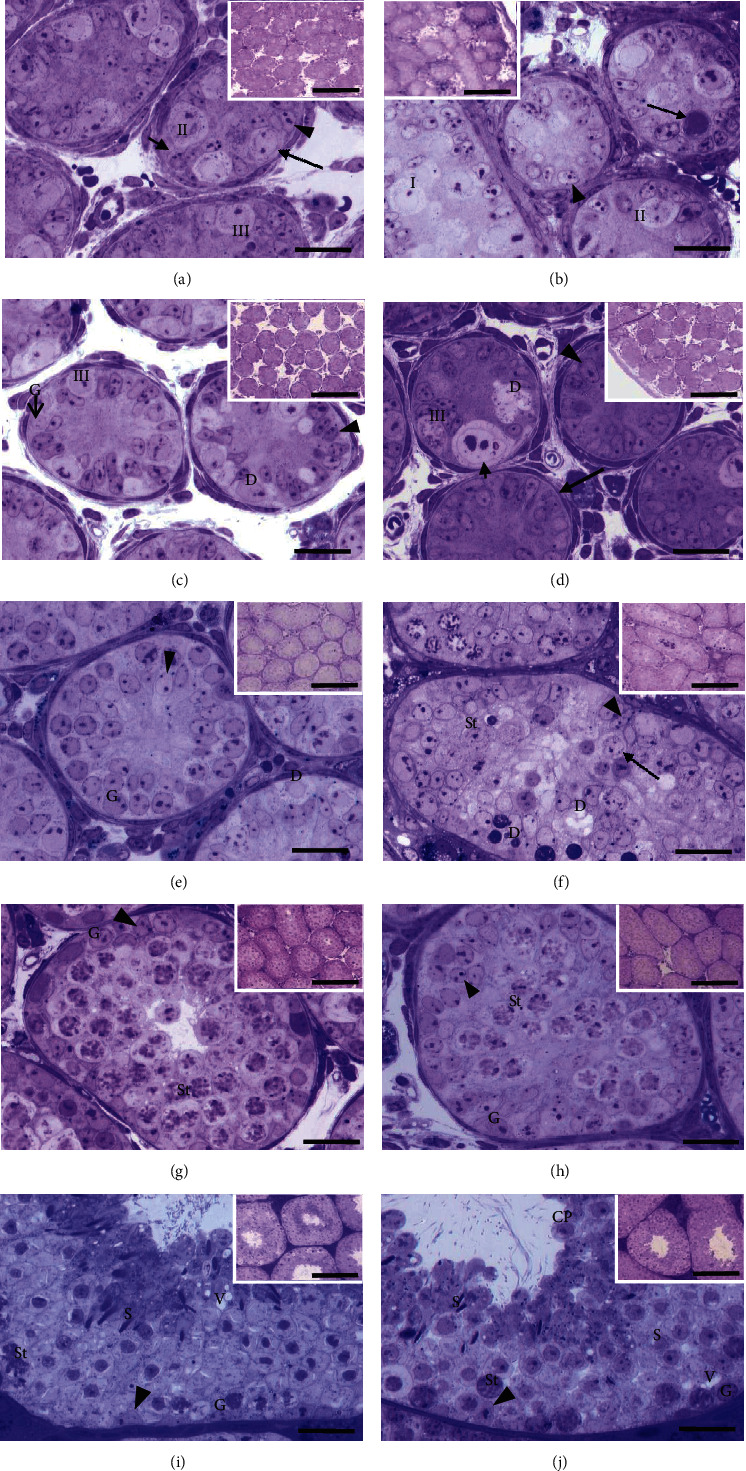
Seminiferous cords/tubules of the testes of control and animals with nicotine administration. (a) Control of 3 dpp, where we could observe subtype II gonocytes with cytoplasmic projections extending toward the basement membrane (short arrow) and subtype III gonocytes in contact with the basement membrane (large arrow) and Sertoli cell. (b) The nicotine group of 3 dpp, where we can appreciate subtype I and II gonocytes (some of which are not in contact with the basement membrane), degenerated gonocytes (arrow), and Sertoli cell. (c) Control of 7 dpp subtype III gonocytes in contact with the basement membrane, type A spermatogonia, and Sertoli cell can be appreciated. (d) The nicotine group of 7 dpp, where we can observe degenerated subtype III gonocytes, giant gonocytes (short arrow), and Sertoli cell. Some cords did not have germ cells (large arrow). (e) Control of 10 dpp; gonocytes are not observed. There are spermatogonia in contact with the basement membrane and a great number of Sertoli cells. (f) Nicotine of 10 dpp. Germ cells without contact with the basement membrane (large arrow); some of these in degeneration (d) and Sertoli cell can be observed. (g) Control of 16 dpp, where we can observe spermatogonia, a great number of pachytene spermatocytes, Sertoli cells, and tubular lumen. (h) The nicotine group of 16 dpp showing spermatogonia and a lower number of spermatocytes without tubular lumen. (i) Control of 35 dpp. A clear spermatogenesis with spermatogonia, spermatocytes, spermatids, Sertoli cell, and mild vacuolization (V) can be observed. (j) The nicotine group of 35 dpp, where we can observe the same characteristics as in (i) and mild cellular peeling. The insertions show the general panorama of the seminiferous cords/tubules. I = subtype I gonocytes; II = subtype II gonocytes; III = subtype III gonocytes; arrowhead = Sertoli cell nucleus; G = spermatogonia; St = spermatocyte; S = spermatids; D = cells in degeneration; V = vacuolization; CP = cellular peeling. Toluidine blue. Bar scale: 20 *μ*m, insertion bar scale: 150 *μ*m.

**Figure 2:**
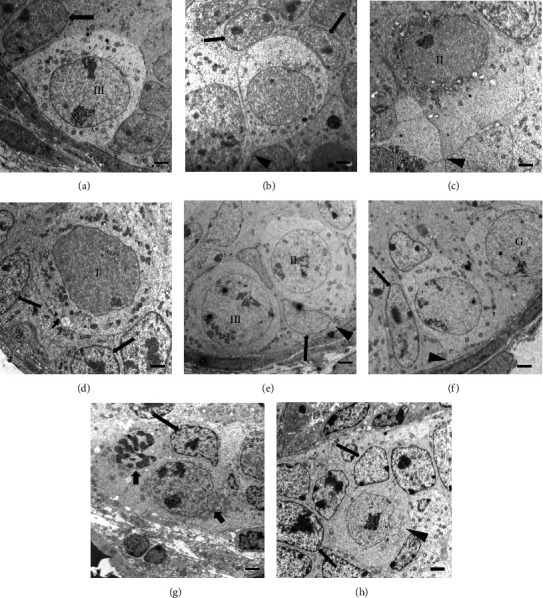
Seminiferous cords of the testes of control and animals with nicotine administration. (a, b) Control of 3 dpp, where we could observe subtype III gonocytes in contact with the basement membrane and in the process of migration to the basement membrane, with cytoplasmic projections extending toward the basal membrane (arrowhead) and Sertoli cell nuclei (arrow). (c, d) The nicotine group of 3 dpp, where we can appreciate subtype I and II gonocytes, some in the process of migration to the basement membrane with cytoplasmic projections extending toward the basement membrane (arrowhead) that could be observed to be in degeneration. The other gonocyte is not in contact with the basement membrane (short arrow). (e, f) Control of 7 dpp, where subtype III gonocytes in contact with the basement membrane and spermatogonia can be appreciated. Cytoplasmic projections extending toward the basement membrane can be observed (arrowhead). (g, h) The nicotine group of 7 dpp, where we can observe degenerated gonocytes (short arrow) and gonocytes without contact with the basement membrane (arrowhead). I = subtype I gonocytes; II = subtype II gonocytes; III = subtype III gonocytes; large arrow = Sertoli cell nucleus, and G = spermatogonia. Electron microscopy. Bar scale: 2 *μ*m.

**Figure 3:**
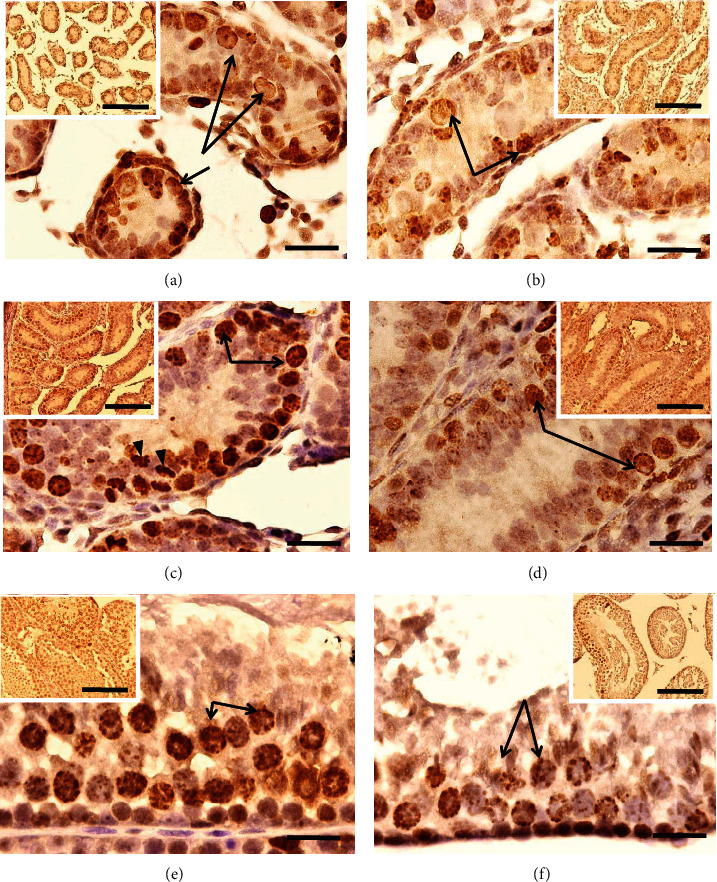
Seminiferous cords/tubules of mice: (a) control of 3 dpp, (b) nicotine group of 3 dpp, (c) control of 10 dpp, (d) nicotine of 10 dpp, (e) control of 35 dpp, and (f) the nicotine group of 35 dpp. A greater number of immunoreactive germ cells to phospho-histone H3 protein can be observed (arrow) in the seminiferous cords/tubules of control animals with respect to the nicotine group. Anaphase (arrowhead). Immunohistochemical study: bar scale: 20 *μ*m, insertion bar scale: 150 *μ*m.

**Figure 4:**
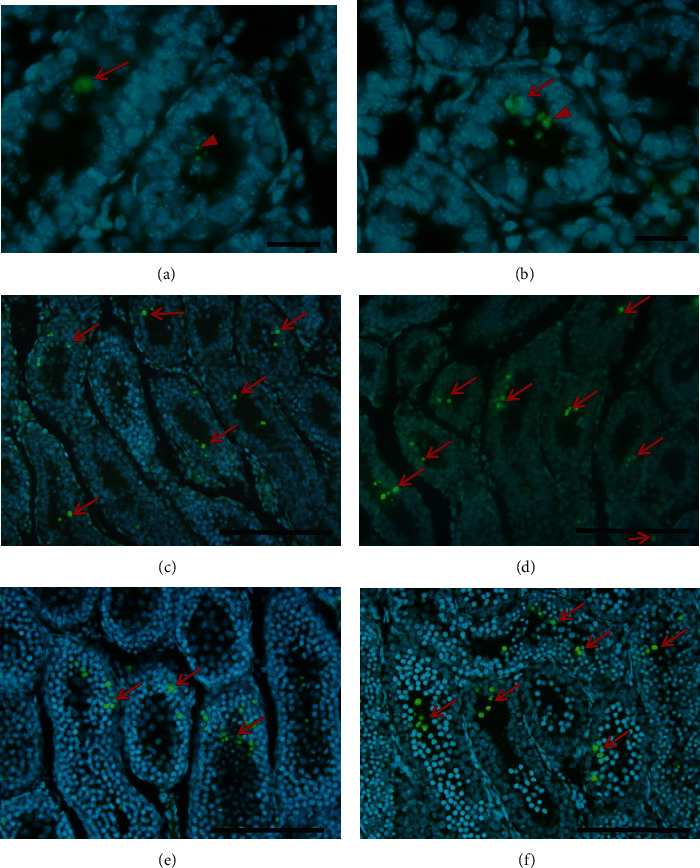
Seminiferous cords/tubules of mice: (a) Control of 3 dpp, (b) the nicotine group of 3 dpp, (c) control of 10 dpp, (d) the nicotine group of 10 dpp, (e) control of 16 dpp, and (f) the nicotine group of 16 dpp. Higher number of cells in apoptosis can be observed (arrow) in the seminiferous cords/tubules of mice with nicotine in comparison with the control group. Also, apoptotic bodies can be appreciated (arrowhead). TUNEL technique contrast with DAPI. (a, b) Bar scale: 20 *μ*m; (c–f) bar scale: 150 *μ*m.

In the control group of 3 dpp; type I, II, and III gonocytes were observed in the central position; and some were in contact with the basement membrane (Figures 1(a), 2(a), and 2(b)). In this group, a major number of gonocytes in proliferation (Figure 3(a)) and the presence of cells in apoptosis (Figure 4(a)) were observed.

In the animals of 3 dpp with nicotine administration, type I and II gonocytes were observed (Figures 1(b), 2(c), and 2(d)). The area of seminiferous cords in comparison with the control group did not show significant difference (*p* > 0.05, Table 1). There was no significant difference in the number of gonocytes without contact with the basement membrane of both groups (*p* > 0.05, Table 2). However, the number of gonocytes in contact with the basement membrane and the number of cells in proliferation were significantly less in the group with nicotine administration (*p* < 0.05, Table 2, Figures 1(b), 2(c), 2(d), and 3(b)) when compared with the control group. The number of cells in apoptosis was increased significantly in the group with nicotine exposure (*p* < 0.05, Table 3, Figure 4(b)).

#### 3.1.2. Animals of 7 dpp

In the control group of 7 dpp (Figures 1(c), 2(e), and 2(f)), type III gonocytes in contact with the basement membrane and type A spermatogonia were seen. In addition, degeneration of some gonocytes was observed. In the nicotine group of this age, type II and III gonocytes and few type A spermatogonia were present. Moreover, degenerated giant gonocytes were observed (Figures 1(d), 2(g), and 2(h)). The area of the seminiferous cords, the number of germ cells in contact with the basement membrane, and the number of spermatogonia showed a significant decrease when compared with the control group (*p* < 0.05, Tables 1 and 2). On the other hand, the number of cells in apoptosis (*p* < 0.05, Table 3) together with gonocytes without contact with the basement membrane was significantly higher in this group (*p* < 0.05, Table 2). Nevertheless, the proliferation showed no significant difference between both groups (*p* > 0.05, Table 2).

#### 3.1.3. Animals of 10 dpp

In the control group of 10 dpp, no type of gonocytes was observed but the presence of spermatogonia A and B and a great number of Sertoli cells, together with cells in degeneration, were detected (Figure 1(e)). In this age, the nicotine group showed a histological structure similar to that of the control group (Figure 1(f)) although with a significantly higher number of cells in apoptosis (*p* < 0.05, Table 3, Figures 4(c) and 4(d)). Degenerated germ cells and gonocytes without contact with the basement membrane were observed in the group exposed to nicotine (Figure 1(f)). The area of seminiferous cords and the number of cells in proliferation and of spermatogonia were significantly less when compared with the control group (*p* < 0.05, Tables 1 and 2, Figures 3(c), 3(d), 4(c), and 4(d)).

#### 3.1.4. Animals of 16 dpp

In the control and nicotine groups of 16 dpp, different types of spermatogonia and a higher number of pachytene spermatocytes were observed. In some seminiferous cords, the lumen began to appear (Figures 1(g) and 1(h)). The area of the seminiferous cords and the number of spermatogonia and of cells in proliferation were significantly less in the nicotine group when compared with control group (*p* < 0.05, Tables 1 and 2). The number of cells in apoptosis did not show significant difference when compared with the control group (*p* > 0.05, Table 3, Figures 4(e) and 4(f)).

Lipoperoxidation (TBARS) did not show significant differences when the nicotinic group was compared with the control group at ages 3, 7, 10, and 16 dpp (*p* > 0.05, Table 3).

#### 3.1.5. Animals of 35 dpp

At 35 dpp, different stages of development of the spermatogenesis, from spermatogonia to elongated spermatids, were observed in both the nicotine and control groups. The histological structure was similar in the two groups, since histological alterations, such as vacuolization and cellular peeling, were present (Figures 1(i) and 1(j)). The area of seminiferous tubules in the nicotine group was significantly less when compared with the control group (*p* < 0.05, Table 1) except at 7 days. The number of cells in apoptosis did not also show significant difference (*p* > 0.05, Table 3); however, the number of spermatogonia and cells in proliferation was less in the nicotine group in comparison with the control (*p* < 0.05, Table 2, Figures 3(e) and 3(f)). Lipoperoxidation (TBARS) was significantly low in the nicotine group when compared with the control (*p* < 0.05, Table 3).

### 3.2. Immunoreactivity (Optical Density) for *α*7-nAChR

**Figure 5:**
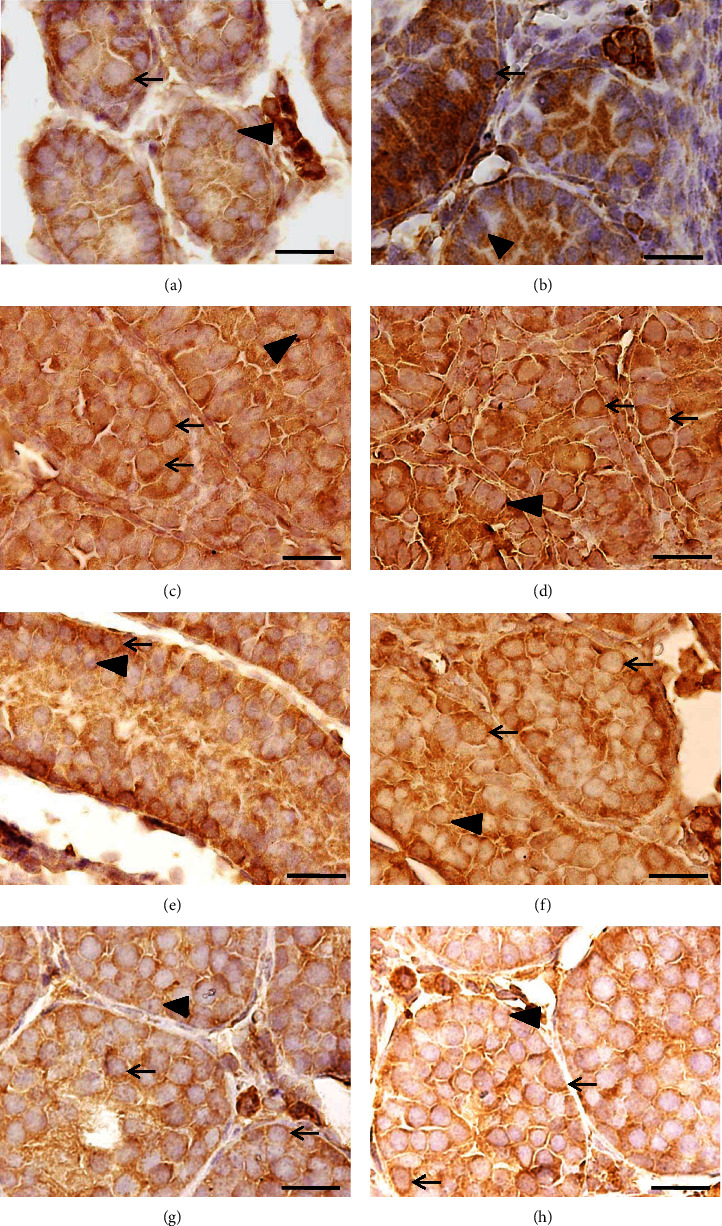
Seminiferous cords/tubules of mice: (a) control of 3 dpp, (b) the nicotine group of 3 dpp, (c) control of 7 dpp, (d) the nicotine group of 7 dpp, (e) control of 10 dpp, (f) the nicotine group of 10 dpp, (g) control of 16 dpp, and (h) the nicotine group of 16 dpp. Immunoreactivity of *α*7-nAChR in germ cells (arrow), as well as in Sertoli cells (arrowhead). Higher OD in germ cells of nicotine groups of 3, 7, and 10 dpp can be observed. In the animals of 16 dpp, the OD between both groups is not different. Immunohistochemical study: bar scale: 20 *μ*m.

According to age, there was *α*7-nAChR immunoreactivity in gonocytes, spermatogonia, and spermatocytes. However, at 3, 7, and 10 dpp, OD was higher in the animals exposed to nicotine (*p* < 0.05). After this age, differences were not observed between both groups (*p* > 0.05, Figures 5(a)–5(h), Table 3).

## 4. Discussion

The consumption of cigarette during pregnancy and lactation in humans and animal models has been demonstrated to have adverse effects on the offspring [5–9, 11–14, 33].

Nicotine, one of the components of cigarette, quickly passes through the placental barrier and to the maternal milk in exposed rats [1, 2]. In this study, the decrease in body weight of the newborn animals in the group exposed to this alkaloid might be related to an indirect effect of this substance, which reduces the availability of oxygen and blood flow to the fetus [34]. This leads to a decrease in birth weight as seen in heavy smokers [35, 36]. A study in rat pups from dams with administration of nicotine at a dose of 0.5 mg/kg, an inferior dose to the one used in this study, reported a significant reduction in body weight [27]. A reduction of 43% in the body weight of offspring proceeding from mice exposed to nicotine in gestation and in the first five days of postnatal life has been reported; nevertheless, these animals recovered their body weight at 35 dpp [37]. In this study, the offspring also recovered their body weight at 35 dpp.

In our nicotine administration protocol, we identified one animal with inguinal bilateral cryptorchidism. With the technique of meta-analysis, a small increase in the risk of cryptorchidism following gestational exposition to cigarette smoke was reported [38]. Other authors found a close relationship between pregnancy and lactational nicotine exposition and development of cryptorchidism. To date, gestational smoking is considered a risk factor for the development of cryptorchidism [39, 40]. It is important to mention that testicular descent comprehends two stages, and the second of them depends on fetal testosterone. It has been reported that nicotine reduces the biosynthesis of testosterone; therefore, this could contribute to the development of cryptorchidism [41].

Alterations in reproduction in sons from mothers who smoked during pregnancy and lactation have been reported [5–9, 11, 33]. Most of the experimental works carried out with nicotine administration in these periods studied the organisms mainly at adult stage [12–16]. Lagunov et al. [12] focused their studies on the effect of *in utero* and lactational exposure to nicotine (1 mg/kg/d s.c.) on the reproductive tract of the offspring and reported histological alterations in the testes at 7 weeks of age. However, this was neither evident at 26 weeks of age nor was the sperm production affected, thus concluding that maternal nicotine exposure can induce transient structural changes in the testis and epididymis of male offspring. On the other hand, Miranda-Spooner et al. [15] reported that the administration of nicotine (2 mg/kg/day) during the same periods did not generate testicular histological alterations at 90 dpp, but in long-term (143 and 196 dpp), there were cellular peeling and epithelial vacuolization. In all the ages studied, they observed abnormalities in the sperm head and tail. Paccola and Miraglia [16] found at 30, 60, and 90 dpp that nicotine exposure (2 mg/kg per day) during intrauterine life and lactation caused intense sloughing of germ cell into the lumen, hence compromising the spermatogenesis in puberty and adulthood; however, these authors did not determine sperm parameters.

Sobinoff et al. [14] demonstrated that maternal cigarette smoke exposure during pregnancy/lactation induces severe neonatal/juvenile germ cell depletion. Aberrant testicular development characterized by abnormal Sertoli and germ cell organization, a depleted spermatogonial stem cell population, atrophic seminiferous tubules, and increased germ cell DNA damage persisted in adult offspring 11 weeks after exposure. These authors also found a reduction in the concentration and sperm motility, as well as an increase in its morphological alterations, thus reducing its fertilization capacity. In spite of the differences in our experimental model, their results, in the short term, coincide with what were reported in this work. Although Sobinoff et al. [14] did not focus on studying the gonocytes, they mentioned degeneration in this type of cell. In the present study, nicotine delayed the maturation of gonocytes to spermatogonia, as demonstrated by the presence of a higher number of gonocytes until 10 dpp.

The mechanism of damage by nicotine that produces lack of maturation of the gonocytes and their degeneration could be explained through different routes: (1) by its indirect action on the hypothalamic-pituitary-testicular axis that modified hormonal production and (2) by direct action on the testicular cells.

In this work, we were not able to determine if nicotine affected the concentrations of testosterone, FSH, or LH. The mechanism of damage by nicotine may involve a direct action on Sertoli cells which have been reported to be altered in laboratory animals with nicotine exposure during pregnancy and lactation [14, 16]. Also, low concentrations of inhibin B in sons of mothers who smoked more than 10 cigarettes per day during pregnancy have been reported [5]. This hormone is produced by Sertoli cells in the testis, and it is positively associated with the function of this type of cell [42]. *In vitro* studies with Sertoli cells from prepubertal animals exposed to nicotine have demonstrated alterations in their functionality (reduced mRNA expression and protein levels of Anti-Mullerian Hormone (AMH) and inhibin B and impaired FSH-r), in addition to downregulation of Bcl2, which is considered a survival factor [43]. Sertoli cell is indispensable in the regulation of gonocyte proliferation for the participation of platelet-derived growth factor- (PDGF-) BB, 17*β*-estradiol (E2), leukemia inhibitory factor (LIF), and retinoic acid (RA) [44], as well as for the maturation of gonocytes to spermatogonia by providing the factors such as RA, PDGF and its receptor, and AMH [19, 45, 46]. In case the gonocytes do not mature, the Sertoli cell provides proapoptotic proteins, such as transforming growth factor-*β* (TGF) and FasL, thus activating pathways such as p53, p21 (WAF1/CIP1), and Bax that are known to participate in the testicle from early stages of development and can be activated by exposure to cytotoxic agents [47–49]. In addition, it has been demonstrated that nicotine acts by reducing mRNA and protein levels of Bcl2, as well as upregulating p53 and caspase-3 mRNA, including protein levels, that adversely affects the germinal epithelium in adult rats [50].

Moreover, nicotine can have direct action on gonocytes. In our study, the presence of higher OD from *α*7-nAChR in the animals exposed to nicotine at 3, 7, and 10 dpp may suggest that the mechanism of action of nicotine is direct on germ cells. Activated nAChR has been shown to increase ion influx, mainly Ca^2+^ [51]. It has been demonstrated that the increase in intracellular calcium of Sertoli cells (TM4) *in vitro* induced mitochondrial membrane depolarization. This produces the release of proapoptotic factors by activating the permeability transition pore and loss of mitochondrial membrane integrity [52–54].

It is likely that during the period of higher OD of *α*7-nAChR in the first three ages studied in this work (3, 7, and 10 dpp), the apoptosis of a part of the gonocyte population took place. This hampered their migration to the basement membrane, which impeded their differentiation and led to a decreased germ cell population in proliferation. This event may have brought about the smaller area in the seminiferous tubules at 35 dpp. This may be reflected in a low volume of ejaculates and sperm count in humans [4, 5], as well as in laboratory animals [14].

On the other hand, it should be considered that exposition to tobacco causes damage to DNA, which accelerates senescence in different organs [55]. In addition to cell cycle arrest, senescent cells secrete an abnormal variety of molecules, including inflammatory cytokines, growth factors, reactive oxygen species (ROS), and extracellular matrix components that modify the cellular microenvironment, which, in turn, causes tissue dysfunction [56]. The testicular tissue develops senescence in elderly animals [57]; however, nicotine has not demonstrated to induce this process in the testicle.

Finally, the mechanism of reproductive damage by nicotine administration during pregnancy and postnatal periods can be through a route different from the theory of oxidative stress associated with nicotine as was postulated [10, 23, 24]. The absence of increases in lipoperoxidation in the nicotine animal groups of 3, 7, 10, and 16 dpp, as well as the reduction in lipoperoxidation at 35 dpp in the nicotine group, could be owed to an excellent testicular antioxidant system at these ages. Also, it could be due to an increase in the activity of enzymes, such as catalase (CAT) and superoxide dismutase (SOD) induced by alkaloid. There are reports that show an increase in the activity of brain SOD, testicular CAT, and testicular glutathione peroxidase (GPx), induced by the administration of different doses of nicotine in different stages of development [58–60]. An increase in the activity of these enzymes may lead to a decrease in the availability of reactive oxygen species.

This work does not include sperm count, serum testosterone, FSH, LH, and inhibin B measurement and antioxidant enzyme activities. These tests may give information on the mechanisms of damage generated by nicotine. Hence, we propose that further studies are necessary to know the mechanisms of damage generated by nicotine on maturation of gonocytes.

## 5. Conclusions

The present study shows that a direct action of the nicotine during pregnancy and postnatal period can alter the process of maturation from gonocytes to spermatogonia and affect the pool of available spermatogonia for spermatogenesis.

## Figures and Tables

**Table 1:** Evaluated parameters (median and interquartile ranges) in control and nicotine-exposed animals.

Age (dpp)	Body weight (g)		Testicular weight/body weight		Blood cotinine (ng/mL)		Area of seminiferous cords/tubules (*μ*m^2^)	
	Control	Nicotine	Control	Nicotine	Control	Nicotine	Control	Nicotine
3	2.360 2.140-2.370	1.990^∗^ 1.770-2.130	0.074 0.042-0.443	0.055 0.047-0.075	0.000 0.000-0.000	1.518^∗^ 0.190-1.660	2402.0 1773.9-3476.9	2573.9 1773.9-3689.9

7	5.250 4.890-5.360	4.490 4.290-5.020	0.0761 0.061-0.093	0.069 0.066-0.079	0.000 0.000-0.000	3.778^∗^ 0.000-5.206	2997.9 2218.0-4236.9	2364.6^∗^ 1466.1-3342.3

10	7.180 6.060-7.420	6.860 5.960-7.750	0.081 0.078-0.094	0.084 0.078-0.090	0.000 0.000-0.000	3.220^∗^ 2.539-4.005	5575 4645-6527	5191.8^∗^ 3943.5-6667.0

16	7.300 7.068-7.640	6.850^∗^ 6.590-7.050	0.260 0.027-0.278	0.143 0.131-0.151	0.000 0.000-0.000	2.015^∗^ 1.015-2.253	9571 6994-13998	7096∗ 5182-9759

35	20.770 17.530-24.020	22.510 10.270-27.260	0.285 0.212-0.333	0.270 0.190-0.315	0.000 0.000-0.000	1.158^∗^ 0.394-3.162	32542 25461-41177	27037^∗^ 21388-37727

^∗^ *p* < 0.05 control vs. nicotine of the same age.

**Table 2:** Evaluated parameters (median and interquartile ranges) in control and nicotine-exposed animals.

Age (dpp)	Number of G in contact with BM/1000 *μ*m^2^		Number of G without contact with BM/1000 *μ*m^2^		Number of spermatogonia/1000 *μ*m^2^		Number of cells in proliferation/1000 *μ*m^2^	
	Control	Nicotine	Control	Nicotine	Control	Nicotine	Control	Nicotine
3	0.978 0.363-2.254	0.534^∗^ 0.273-1.352	0.416 0.343-1.156	0.654 0.307-1.515			10.380 6.817-14.000	6.072^∗^ 2.539-9.539

7	1.645 0.708-3.606	0.859^∗^ 0.328-1.926	0.010 0.010-0.343	0.409^∗^ 0.353-0.873	0.144 0.123-0.364	0.108^∗^ 0.088-0.140	1.1228 0.324-1.868	1.074 0.726-1.7462

10			0.010 0.010-0.612	0.199^∗^ 0.124-0.406	1.291 1.003-1.665	1.066^∗^ 0.398-1.701	5.122 1.396-8.267	3.162^∗^ 0.317-8.605

16					1.712 0.937-3.210	1.378^∗^ 1.005-1.761	2.114 1.965-2.787	1.784^∗^ 1.093-2.290

35					0.912 0.524-1.489	0.517^∗^ 0.216-0.892	6.809 5.157-9.583	5.040^∗^ 3.102-7.630

^∗^ *p* < 0.05 control vs. nicotine of the same age. G = gonocyte; BM = basement membrane.

**Table 3:** Evaluated parameters (median and interquartile ranges) in control and nicotine-exposed animals.

Age (dpp)	Number of cells in apoptosis/1000 *μ*m^2^		TBARS (nmoles of TBARS per mg of protein)		OD of *α*7-nAChR (arbitrary units/10 *μ*m^2^)	
	Control	Nicotine	Control	Nicotine	Control	Nicotine
3	0.351 0.174-1.847	0.825^∗^ 0.583-1.847	0.625 0.433-0.841	0.607 0.310-0.834	0.163 0.035-1.464	0.326^∗^ 0.135-7.016

7	0.439 0.124-2.150	1.126^∗^ 0.199-2.265	0.625 0.433-0.841	0.607 0.310-0.834	0.208 0.063-0.250	0.290^∗^ 0.118-0.575

10	0.692 0.128-2.158	1.197^∗^ 0.185-4.183	0.625 0.433-0.841	0.607 0.310-0.834	0.142 0.034-0.244	0.276^∗^ 0.111-0.535

16	0.329 0.042-5.723	0.332 0.053-2.947	0.625 0.433-0.841	0.607 0.310-0.834	0.164 0.038-0.280	0.111 0.003-0.263

35	0.199 0.048-0.925	0.136 0.030-0.687	0.355 0.209-0.403	0.097^∗^ 0.065-0.174	0.098 0.089-0.269	0.1458 0.102-0.165

^∗^ *p* < 0.05 control vs. nicotine of the same age.

## Data Availability

All data used to support the findings of this study are included within the supplementary information file.

## Supplementary Materials

Supplementary Materials The data used to support the findings of this study are included within the supplementary information.
