# Supplementary material for: Fetal and Postnatal Nicotine Exposure Modifies Maturation of Gonocytes to Spermatogonia in Mice
Source: Anal Cell Pathol (Amst). 2020 Dec 15;2020:8892217. doi: 10.1155/2020/8892217 (PMC7758125; doi:10.1155/2020/8892217)
Supplement: Supplementary Materials — The data used to support the findings of this study are included within the supplementary information. [file 8892217.f1.docx]

**DATA IN BRIEF**

**D= days**

**C= Control**

**N= nicotine**

**Area of seminiferous**

**cords/ tubules (µm^2^)**

**Descriptive Statistics: 3D C, 3D N, 7D C, 7D N, 10D C, 10D N, 16D C, 16D N, 35D C, 35D N**

Variable N N* Mean SE Mean StDev Minimum Q1 Median Q3 Maximum

3D C 34 0 2406.3 65.2 380.0 1773.9 2164.4 2402.0 2721.6 3476.9

3D N 62 0 2604.1 60.6 477.4 1773.9 2209.0 2573.9 2997.9 3659.9

7D C 67 0 2986.7 40.3 330.2 2218.0 2751.7 2997.9 3211.3 4236.9

7D N 109 0 2375.8 36.2 378.2 1466.1 2111.1 2364.6 2652.2 3342.8

10D C 15 0 5551 131 509 4645 5348 5575 5982 6527

10D N 53 0 5164.9 88.5 644.1 3943.5 4763.0 5191.8 5582.9 6677.0

16D C 43 0 9369 317 2078 70 8304 9533 10218 13998

16D N 23 0 7020 225 1080 5182 6101 7096 7488 9759

35D C 28 0 32700 801 4237 25461 28874 32542 35207 41177

35D N 11 0 27419 1553 5152 21388 23285 27037 31363 37727

**Mann-Whitney Test and CI: 3D C, 3D N**

N Median

3D C 34 2402.0

3D N 62 2573.9

Point estimate for η1 - η2 is -186.9

95.1 Percent CI for η1 - η2 is (-389.9,0.1)

W = 1404.5

Test of η1 = η2 vs η1 ≠ η2 is significant at 0.0616

The test is significant at 0.0614 (adjusted for ties)

**Mann-Whitney Test and CI: 7D C, 7D N**

N Median

7D C 67 2997.9

7D N 109 2364.6

Point estimate for η1 - η2 is 614.6

95.0 Percent CI for η1 - η2 is (505.1,721.4)

W = 8764.5

Test of η1 = η2 vs η1 ≠ η2 is significant at 0.0000

The test is significant at 0.0000 (adjusted for ties)

**Mann-Whitney Test and CI: 10D C, 10D N**

N Median

10D C 15 5574.7

10D N 53 5191.8

Point estimate for η1 - η2 is 411.3

95.1 Percent CI for η1 - η2 is (35.0,738.7)

W = 665.0

Test of η1 = η2 vs η1 ≠ η2 is significant at 0.0297

The test is significant at 0.0297 (adjusted for ties)

**Mann-Whitney Test and CI: 16D C, 35D C**

N Median

16D C 43 9533

35D C 28 32542

Point estimate for η1 - η2 is -23307

95.1 Percent CI for η1 - η2 is (-24893,-21655)

W = 946.0

Test of η1 = η2 vs η1 ≠ η2 is significant at 0.0000

The test is significant at 0.0000 (adjusted for ties)

**Mann-Whitney Test and CI: 35D C, 35D N**

N Median

35D C 28 32542

35D N 11 27037

Point estimate for η1 - η2 is 5396

95.2 Percent CI for η1 - η2 is (2109,9189)

W = 648.0

Test of η1 = η2 vs η1 ≠ η2 is significant at 0.0063

**BLOOD COTININE (NG/ML)**

**Descriptive Statistics: control 3d, nicot 3d, control 7d, nicotin 7d, control 10d, ...**

Variable N N* Mean SE Mean StDev Minimum Q1 Median Q3

control 3d 4 0 0.000008 0.000002 0.000005 0.000001 0.000003 0.000010 0.000010

nicot 3d 5 0 1.091 0.303 0.677 0.190 0.364 1.518 1.605

control 7d 8 0 0.000009 0.000001 0.000004 0.000000 0.000010 0.000010 0.000010

nicotin 7d 7 0 3.406 0.651 1.721 0.000 2.576 3.778 4.631

control 10d 4 0 0.000003 0.000002 0.000005 0.000000 0.000000 0.000000 0.000008

nicotin 10d 3 0 3.271 0.410 0.709 2.589 2.589 3.220 4.005

control 16d 6 0 0.000008 0.000002 0.000004 0.000000 0.000008 0.000010 0.000010

nicotin 16d 5 0 1.767 0.247 0.552 1.015 1.184 2.015 2.226

control 35d 5 0 0.000008 0.000002 0.000004 0.000000 0.000005 0.000010 0.000010

nicotin 35 d 7 0 1.301 0.332 0.878 0.394 0.802 1.158 1.329

Variable Maximum

control 3d 0.000010

nicot 3d 1.660

control 7d 0.000010

nicotin 7d 5.206

control 10d 0.000010

nicotin 10d 4.005

control 16d 0.000010

nicotin 16d 2.253

control 35d 0.000010

nicotin 35 d 3.162

**Mann-Whitney Test and CI: control 3d, nicot 3d**

N Median

control 3d 4 0.000

nicot 3d 5 1.518

Point estimate for η1 - η2 is -1.518

96.3 Percent CI for η1 - η2 is (-1.660,-0.190)

W = 10.0

Test of η1 = η2 vs η1 ≠ η2 is significant at 0.0200

The test is significant at 0.0151 (adjusted for ties)

**Mann-Whitney Test and CI: control 7d, nicotin 7d**

N Median

control 7d 8 0.000

nicotin 7d 7 3.778

Point estimate for η1 - η2 is -3.778

95.7 Percent CI for η1 - η2 is (-4.631,-2.576)

W = 40.0

Test of η1 = η2 vs η1 ≠ η2 is significant at 0.0065

The test is significant at 0.0022 (adjusted for ties)

**Mann-Whitney Test and CI: control 10d, nicotin 10d**

N Median

control 10d 4 0.000

nicotin 10d 3 3.220

Point estimate for η1 - η2 is -3.220

94.8 Percent CI for η1 - η2 is (-4.005,-2.589)

W = 10.0

Test of η1 = η2 vs η1 ≠ η2 is significant at 0.0518

The test is significant at 0.0319 (adjusted for ties)

**Mann-Whitney Test and CI: control 16d, nicotin 16d**

N Median

control 16d 6 0.0000

nicotin 16d 5 2.0150

Point estimate for η1 - η2 is -2.0150

96.4 Percent CI for η1 - η2 is (-2.2531,-1.0149)

W = 21.0

Test of η1 = η2 vs η1 ≠ η2 is significant at 0.0081

The test is significant at 0.0055 (adjusted for ties)

**Mann-Whitney Test and CI: control 35d, nicotin 35 d**

N Median

control 35d 5 0.000

nicotin 35 d 7 1.158

Point estimate for η1 - η2 is -1.158

96.5 Percent CI for η1 - η2 is (-3.162,-0.394)

W = 15.0

Test of η1 = η2 vs η1 ≠ η2 is significant at 0.0058

The test is significant at 0.0042 (adjusted for ties)

TBARS (nmoles of TBARS per mg of protein)

**Descriptive Statistics: c, n**

Variable N N* Mean SE Mean StDev Minimum Q1 Median Q3 Maximum

c 5 0 0.6090 0.0721 0.1612 0.4331 0.4577 0.6259 0.7518 0.8410

n 4 0 0.589 0.108 0.215 0.310 0.381 0.607 0.781 0.834

**Mann-Whitney Test and CI: c, n**

N Median

c 5 0.6259

n 4 0.6072

Point estimate for η1 - η2 is 0.0194

96.3 Percent CI for η1 - η2 is (-0.3516,0.3531)

W = 27.0

Test of η1 = η2 vs η1 ≠ η2 is significant at 0.7133

c n Due to the reduced size of the testes of 3, 7, 10 and 16 dpp, they were homogenized in groups of nicotine and control per each age.

0.841045 0.834043

0.625946 0.594144

0.662591 0.620219

0.482434 0.309578

0.433066

**Descriptive Statistics: c, n de 35 dpp**

Variable N N* Mean SE Mean StDev Minimum Q1 Median Q3 Maximum

c 4 0 0.3309 0.0433 0.0866 0.2098 0.2395 0.3552 0.3980 0.4033

n 7 0 0.1118 0.0158 0.0419 0.0659 0.0678 0.0978 0.1568 0.1744

**Mann-Whitney Test and CI: c, n**

N Median

c 4 0.3552

n 7 0.0978

Point estimate for η1 - η2 is 0.2309

95.3 Percent CI for η1 - η2 is (0.1121,0.3143)

W = 38.0

Test of η1 = η2 vs η1 ≠ η2 is significant at 0.0107

c n 35 DPP

0.209848 0.174430

0.403319 0.156790

0.382048 0.067787

0.328289 0.096933

0.123187

0.097804

0.065907

**OD OF Α 7 CHRH**

**(ARBITRARY UNITS/10µm^2^)**

**Mann-Whitney Test and CI: ctrl 7d, nicot 7d**

N Median

ctrl 7d 6 0.2087

nicot 7d 18 0.2909

Point estimate for η1 - η2 is -0.1229

95.1 Percent CI for η1 - η2 is (-0.2157,-0.0212)

W = 39.0

Test of η1 = η2 vs η1 ≠ η2 is significant at 0.0179

**Mann-Whitney Test and CI: ctl 10 d, nicot 10 d**

N Median

ctl 10 d 7 0.1421

nicot 10 d 13 0.2763

Point estimate for η1 - η2 is -0.1596

95.2 Percent CI for η1 - η2 is (-0.2781,-0.0692)

W = 37.0

Test of η1 = η2 vs η1 ≠ η2 is significant at 0.0043

**Mann-Whitney Test and CI: ctrl 16 d, nico 16 d**

N Median

ctrl 16 d 5 0.1645

nico 16 d 7 0.1119

Point estimate for η1 - η2 is 0.0526

96.5 Percent CI for η1 - η2 is (-0.0757,0.1962)

W = 39.0

Test of η1 = η2 vs η1 ≠ η2 is significant at 0.3299

**Mann-Whitney Test and CI: ctrl 35 d, nicot 35 d**

N Median

ctrl 35 d 5 0.0987

nicot 35 d 7 0.1459

Point estimate for η1 - η2 is -0.0177

96.5 Percent CI for η1 - η2 is (-0.0690,0.1064)

W = 26.0

Test of η1 = η2 vs η1 ≠ η2 is significant at 0.3299

**Mann-Whitney Test and CI: ni 3d, ctl 3d**

N Median

ni 3d 10 0.326

ctl 3d 13 0.163

Point estimate for η1 - η2 is 0.158

95.6 Percent CI for η1 - η2 is (0.030,1.200)

W = 158.0

Test of η1 = η2 vs η1 ≠ η2 is significant at 0.0200

Variable N N* Mean SE Mean StDev Minimum Q1 Median Q3 Maximum

ni 3d 10 0 1.722 0.886 2.803 0.135 0.234 0.326 2.767 7.016

ctl 3d 13 0 0.303 0.109 0.395 0.035 0.109 0.163 0.268 1.464

ctrl 7d 6 0 0.1793 0.0339 0.0831 0.0637 0.0876 0.2087 0.2493 0.2500

nicot 7d 18 0 0.3029 0.0268 0.1138 0.1184 0.2320 0.2909 0.3810 0.5758

ctl 10 d 7 0 0.1356 0.0264 0.0697 0.0345 0.0886 0.1421 0.1912 0.2446

nicot 10 d 13 0 0.3001 0.0339 0.1223 0.1110 0.2277 0.2763 0.3739 0.5353

ctrl 16 d 5 0 0.1564 0.0438 0.0979 0.0380 0.0606 0.1645 0.2481 0.2801

nico 16 d 7 0 0.0985 0.0335 0.0888 0.0036 0.0198 0.1119 0.1337 0.2638

ctrl 35 d 5 0 0.1416 0.0341 0.0761 0.0896 0.0924 0.0987 0.2122 0.2693

nicot 35 d 7 0 0.14130 0.00951 0.02516 0.10257 0.11638 0.14586 0.16425 0.16522

**NUMBER OF CELLS IN PROLIFERATION/ 1000µm^2^**

**Descriptive Statistics: 3C, 3N, 7C, 7N, 10C, 10N, 16C, 16N, 35C, 35N**

Variable N N* Mean SE Mean StDev Minimum Q1 Median Q3 Maximum

3C 17 0 10.667 0.573 2.361 6.817 8.283 10.380 12.895 14.000

3N 63 0 6.139 0.202 1.606 2.539 4.974 6.072 7.364 9.539

7C 39 0 1.0675 0.0624 0.3894 0.3244 0.7061 1.1228 1.3526 1.8684

7N 27 0 1.1365 0.0500 0.2599 0.7268 0.9797 1.0745 1.2889 1.7462

10C 49 0 5.177 0.237 1.661 1.396 4.028 5.122 6.346 8.267

10N 60 0 3.288 0.225 1.740 0.317 2.188 3.162 4.127 8.605

16C 5 0 2.337 0.182 0.407 1.965 2.007 2.114 2.779 2.787

16N 14 0 1.759 0.102 0.380 1.093 1.451 1.784 2.052 2.290

35C 19 0 6.727 0.261 1.138 5.157 5.667 6.809 7.608 9.583

35N 15 0 5.248 0.354 1.371 3.102 4.324 5.040 6.329 7.630

**Mann-Whitney Test and CI: 3C, 3N**

N Median

3C 17 10.380

3N 63 6.072

Point estimate for η1 - η2 is 4.549

95.0 Percent CI for η1 - η2 is (3.244,5.852)

W = 1168.0

Test of η1 = η2 vs η1 ≠ η2 is significant at 0.0000

**Mann-Whitney Test and CI: 7C, 7N**

N Median

7C 39 1.1228

7N 27 1.0745

Point estimate for η1 - η2 is -0.0745

95.1 Percent CI for η1 - η2 is (-0.2520,0.1221)

W = 1247.5

Test of η1 = η2 vs η1 ≠ η2 is significant at 0.4455

The test is significant at 0.4454 (adjusted for ties)

**Mann-Whitney Test and CI: 10C, 10N**

N Median

10C 49 5.122

10N 60 3.162

Point estimate for η1 - η2 is 2.054

95.1 Percent CI for η1 - η2 is (1.326,2.676)

W = 3554.0

Test of η1 = η2 vs η1 ≠ η2 is significant at 0.0000

**Mann-Whitney Test and CI: 35C, 35N**

N Median

35C 19 6.809

35N 15 5.040

Point estimate for η1 - η2 is 1.467

95.2 Percent CI for η1 - η2 is (0.533,2.486)

W = 420.0

Test of η1 = η2 vs η1 ≠ η2 is significant at 0.0025

**Number of spermatogonia/ 1000µm^2^**

**Descriptive Statistics: 7c, 7n, 10c, 10n, 16c, 16n, 35c, 35n**

Variable N N* Mean SE Mean StDev Minimum Q1 Median Q3 Maximum

7c 6 0 0.1831 0.0374 0.0916 0.1236 0.1304 0.1440 0.2336 0.3641

7n 10 0 0.10949 0.00440 0.01391 0.08855 0.10147 0.10802 0.11537 0.14099

10c 9 0 1.3258 0.0825 0.2474 1.0030 1.0932 1.2917 1.6000 1.6653

10n 44 0 1.0894 0.0441 0.2926 0.3981 0.8926 1.0661 1.3061 1.7012

16c 42 0 1.787 0.106 0.688 0.937 1.155 1.712 2.332 3.210

16n 25 0 1.3559 0.0508 0.2541 1.0058 1.0808 1.3789 1.6246 1.7616

35c 22 0 0.9803 0.0754 0.3537 0.5244 0.6465 0.9122 1.3932 1.4893

35n 10 0 0.5205 0.0571 0.1806 0.2160 0.4118 0.5177 0.6149 0.8928

**Mann-Whitney Test and CI: 7c, 7n**

N Median

7c 6 0.1440

7n 10 0.1080

Point estimate for η1 - η2 is 0.0370

95.5 Percent CI for η1 - η2 is (0.0207,0.0899)

W = 79.0

Test of η1 = η2 vs η1 ≠ η2 is significant at 0.0029

**Mann-Whitney Test and CI: 10c, 10n**

N Median

10c 9 1.2917

10n 44 1.0661

Point estimate for η1 - η2 is 0.2368

95.2 Percent CI for η1 - η2 is (0.0158,0.4511)

W = 333.0

Test of η1 = η2 vs η1 ≠ η2 is significant at 0.0340

**Mann-Whitney Test and CI: 16c, 16n**

N Median

16c 42 1.7120

16n 25 1.3789

Point estimate for η1 - η2 is 0.2944

95.0 Percent CI for η1 - η2 is (0.0251,0.6863)

W = 1598.5

Test of η1 = η2 vs η1 ≠ η2 is significant at 0.0275

The test is significant at 0.0275 (adjusted for ties)

**Mann-Whitney Test and CI: 35c, 35n**

N Median

35c 22 0.9122

35n 10 0.5177

Point estimate for η1 - η2 is 0.4076

95.1 Percent CI for η1 - η2 is (0.1757,0.7712)

W = 452.0

Test of η1 = η2 vs η1 ≠ η2 is significant at 0.0003

**Number of cells in apoptosis/ 1000*μ*m^2^**

**Descriptive Statistics: 3 C, 3 N, 7 C, 7 N, 10 C, 10 N, 16 C, 16 N, 35 C, 35 N**

Error

estándar

de la

Variable N N* Media media Desv.Est. Mediana

3 C 16 0 0.556 0.116 0.462 0.351

3 N 24 0 0.9508 0.0758 0.3714 0.8257

7 C 40 0 0.5456 0.0642 0.4062 0.4392

7 N 40 0 1.0811 0.0622 0.3935 1.1264

10 C 28 0 0.805 0.103 0.544 0.692

10 N 51 0 1.1941 0.0882 0.6299 1.1972

16 C 77 0 0.5733 0.0873 0.7664 0.3290

16 N 102 0 0.4799 0.0479 0.4836 0.3327

35 C 33 0 0.2661 0.0357 0.2052 0.1994

35 N 26 0 0.1778 0.0293 0.1494 0.1368

**Mann-Whitney Test and CI: 3 C, 3 N**

N Mediana

3 C 16 0.3509

3 N 24 0.8257

La estimación del punto para η1 - η2 es -0.4621

95.2 El porcentaje IC para η1 - η2 es (-0.5923,-0.2296)

W = 216.0

Prueba de η1 = η2 vs. η1 ≠ η2 es significativa en 0.0021

**Mann-Whitney Test and CI: 7 C, 7 N**

N Mediana

7 C 40 0.4392

7 N 40 1.1264

La estimación del punto para η1 - η2 es -0.5657

95.1 El porcentaje IC para η1 - η2 es (-0.7500,-0.4048)

W = 1062.0

Prueba de η1 = η2 vs. η1 ≠ η2 es significativa en 0.0000

**Mann-Whitney Test and CI: 10 C, 10 N**

N Mediana

10 C 28 0.6918

10 N 51 1.1972

La estimación del punto para η1 - η2 es -0.4221

95.0 El porcentaje IC para η1 - η2 es (-0.6118,-0.1659)

W = 796.0

Prueba de η1 = η2 vs. η1 ≠ η2 es significativa en 0.0009

**Mann-Whitney Test and CI: 16 C, 16 N**

N Mediana

16 C 77 0.3290

16 N 102 0.3327

La estimación del punto para η1 - η2 es 0.0246

95.0 El porcentaje IC para η1 - η2 es (-0.0553,0.1053)

W = 7125.0

Prueba de η1 = η2 vs. η1 ≠ η2 es significativa en 0.5709

**Mann-Whitney Test and CI: 35 C, 35 N**

N Mediana

35 C 33 0.1994

35 N 26 0.1368

La estimación del punto para η1 - η2 es 0.0588

95.0 El porcentaje IC para η1 - η2 es (-0.0033,0.1357)

W = 1108.0

Prueba de η1 = η2 vs. η1 ≠ η2 es significativa en 0.0728

**Number of G in contact with BM/ 1000µm^2^**

**Descriptive Statistics: C1, C2, C3, C4**

Error

estándar

de la

Variable N N* Media media Desv.Est. Q1 Mediana

C1 34 0 1.0095 0.0862 0.5028 0.5464 0.9783

C2 41 0 0.6266 0.0453 0.2897 0.3885 0.5342

C3 54 0 1.6866 0.0779 0.5724 1.2584 1.6450

C4 101 0 0.8781 0.0383 0.3852 0.5033 0.8593

**Mann-Whitney Test and CI: C1, C2**

N Mediana

C1 34 0.9783

C2 41 0.5342

La estimación del punto para η1 - η2 es 0.3421

95.0 El porcentaje IC para η1 - η2 es (0.1240,0.5478)

W = 1626.5

Prueba de η1 = η2 vs. η1 ≠ η2 es significativa en 0.0004

La prueba es significativa en 0.0004 (ajustado por empates)

**Mann-Whitney Test and CI: C3, C4**

N Mediana

C3 54 1.6450

C4 101 0.8593

La estimación del punto para η1 - η2 es 0.7812

95.0 El porcentaje IC para η1 - η2 es (0.6113,0.9492)

W = 6307.0

Prueba de η1 = η2 vs. η1 ≠ η2 es significativa en 0.0000

La prueba es significativa en 0.0000 (ajustado por empates)

**Number of G without contact with BM/ 1000µm^2^**

**Descriptive Statistics: C1, C2, C3, C4, C5, C6**

Error

estándar

de la

Variable N N* Media media Desv.Est. Q1 Mediana

C1 11 0 0.5654 0.0897 0.2975 0.3915 0.4163

C2 30 0 0.6892 0.0549 0.3008 0.4390 0.6545

C3 16 0 0.0896 0.0357 0.1427 0.0100 0.0100

C4 7 0 0.5553 0.0851 0.2253 0.3582 0.4099

C5 10 0 0.0703 0.0603 0.1906 0.0100 0.0100

C6 8 0 0.2375 0.0362 0.1024 0.1803 0.1993

**Mann-Whitney Test and CI: C1, C2**

N Mediana

C1 11 0.4163

C2 30 0.6545

La estimación del punto para η1 - η2 es -0.1132

95.3 El porcentaje IC para η1 - η2 es (-0.3797,0.0759)

W = 190.0

Prueba de η1 = η2 vs. η1 ≠ η2 es significativa en 0.2334

La prueba es significativa en 0.2332 (ajustado por empates)

**Mann-Whitney Test and CI: C3, C4**

N Mediana

C3 16 0.0100

C4 7 0.4099

La estimación del punto para η1 - η2 es -0.3967

95.1 El porcentaje IC para η1 - η2 es (-0.6720,-0.3431)

W = 136.0

Prueba de η1 = η2 vs. η1 ≠ η2 es significativa en 0.0002

La prueba es significativa en 0.0001 (ajustado por empates)

**Mann-Whitney Test and CI: C5, C6**

N Mediana

C5 10 0.0100

C6 8 0.1993

La estimación del punto para η1 - η2 es -0.1814

95.4 El porcentaje IC para η1 - η2 es (-0.3773,-0.1695)

W = 63.0

Prueba de η1 = η2 vs. η1 ≠ η2 es significativa en 0.0051

La prueba es significativa en 0.0028 (ajustado por empates)

**TESTICULAR WEIGHT/ BODY WEIGHT**

**Mann-Whitney Test and CI: C1, C2**

N Mediana

C1 5 0.0748

C2 6 0.0551

La estimación del punto para η1 - η2 es 0.0102

96.4 El porcentaje IC para η1 - η2 es (-0.0225,0.3809)

W = 32.0

Prueba de η1 = η2 vs. η1 ≠ η2 es significativa en 0.7842

La prueba es significativa en 0.7837 (ajustado por empates)

**Mann-Whitney Test and CI: C3, C4**

N Mediana

C3 5 0.07619

C4 5 0.06993

La estimación del punto para η1 - η2 es 0.00626

96.3 El porcentaje IC para η1 - η2 es (-0.00859,0.02336)

W = 31.0

Prueba de η1 = η2 vs. η1 ≠ η2 es significativa en 0.5309

**Mann-Whitney Test and CI: C5, C6**

N Mediana

C5 4 0.08169

C6 4 0.08439

La estimación del punto para η1 - η2 es -0.00048

97.0 El porcentaje IC para η1 - η2 es (-0.01187,0.01565)

W = 17.0

Prueba de η1 = η2 vs. η1 ≠ η2 es significativa en 0.8852

**Mann-Whitney Test and CI: C7, C8**

N Mediana

C7 5 0.2603

C8 5 0.1431

La estimación del punto para η1 - η2 es 0.1172

96.3 El porcentaje IC para η1 - η2 es (-0.1156,0.1363)

W = 35.0

Prueba de η1 = η2 vs. η1 ≠ η2 es significativa en 0.1437

**Mann-Whitney Test and CI: C9, C10**

N Mediana

C9 6 0.28505

C10 5 0.26999

La estimación del punto para η1 - η2 es 0.02047

96.4 El porcentaje IC para η1 - η2 es (-0.04052,0.09391)

W = 43.0

Prueba de η1 = η2 vs. η1 ≠ η2 es significativa en 0.2353

**Descriptive Statistics: C1, C2, C3, C4, C5, C6, C7, C8, C9, C10**

Error

estándar de

Variable N N* Media la media Desv.Est. Mediana

C1 5 0 0.1370 0.0769 0.1720 0.0748

C2 6 0 0.05799 0.00482 0.01182 0.05511

C3 5 0 0.07741 0.00510 0.01141 0.07619

C4 5 0 0.07295 0.00282 0.00631 0.06993

C5 4 0 0.08410 0.00358 0.00717 0.08169

C6 4 0 0.08450 0.00264 0.00527 0.08439

C7 5 0 0.2181 0.0480 0.1073 0.2603

C8 5 0 0.14324 0.00346 0.00773 0.14306

C9 6 0 0.2858 0.0170 0.0417 0.2851

C10 5 0 0.2622 0.0208 0.0464 0.2700

**BODY WEIGHT**

**(gr)**

**Mann-Whitney Test and CI: C1, C2**

N Mediana

C1 5 0.00160

C2 6 0.00110

La estimación del punto para η1 - η2 es 0.00030

96.4 El porcentaje IC para η1 - η2 es (-0.00040,0.00920)

W = 38.5

Prueba de η1 = η2 vs. η1 ≠ η2 es significativa en 0.1441

La prueba es significativa en 0.1414 (ajustado por empates)

**Mann-Whitney Test and CI: C3, C4**

N Mediana

C3 5 0.00400

C4 5 0.00300

La estimación del punto para η1 - η2 es 0.00100

96.3 El porcentaje IC para η1 - η2 es (-0.00000,0.00200)

W = 33.5

Prueba de η1 = η2 vs. η1 ≠ η2 es significativa en 0.2506

La prueba es significativa en 0.2040 (ajustado por empates)

**Mann-Whitney Test and CI: C5, C6**

N Mediana

C5 4 0.00575

C6 4 0.00570

La estimación del punto para η1 - η2 es 0.00005

97.0 El porcentaje IC para η1 - η2 es (-0.00200,0.00210)

W = 18.5

Prueba de η1 = η2 vs. η1 ≠ η2 es significativa en 1.0000

La prueba es significativa en 1.0000 (ajustado por empates)

**Mann-Whitney Test and CI: C7, C8**

N Mediana

C7 5 0.01845

C8 5 0.01000

La estimación del punto para η1 - η2 es 0.00845

96.3 El porcentaje IC para η1 - η2 es (-0.00800,0.01115)

W = 35.0

Prueba de η1 = η2 vs. η1 ≠ η2 es significativa en 0.1437

La prueba es significativa en 0.1314 (ajustado por empates)

**Mann-Whitney Test and CI: C9, C10**

N Mediana

C9 6 0.06050

C10 5 0.06900

La estimación del punto para η1 - η2 es -0.00675

96.4 El porcentaje IC para η1 - η2 es (-0.01501,0.03950)

W = 30.0

Prueba de η1 = η2 vs. η1 ≠ η2 es significativa en 0.3153
